# The Impact of Applying Quality Management Practices on Patient Centeredness in Jordanian Public Hospitals: Results of Predictive Modeling

**DOI:** 10.1177/0046958018754739

**Published:** 2018-02-26

**Authors:** Heba H. Hijazi, Heather L. Harvey, Mohammad S. Alyahya, Hussam A. Alshraideh, Rabah M. Al abdi, Sanjai K. Parahoo

**Affiliations:** 1 Jordan University of Science and Technology, Irbid, Jordan; 2 The Hashemite University, Zarqa, Jordan; 3 Hamdan Bin Mohammed Smart University, Dubai, UAE

**Keywords:** patient-centered care, accreditation, quality improvement, quality management, patient satisfaction, leadership, Jordan

## Abstract

Targeting the patient’s needs and preferences has become an important contributor for improving care delivery, enhancing patient satisfaction, and achieving better clinical outcomes. This study aimed to examine the impact of applying quality management practices on patient centeredness within the context of health care accreditation and to explore the differences in the views of various health care workers regarding the attributes affecting patient-centered care. Our study followed a cross-sectional survey design wherein 4 Jordanian public hospitals were investigated several months after accreditation was obtained. Total 829 clinical/nonclinical hospital staff members consented for study participation. This sample was divided into 3 main occupational categories to represent the administrators, nurses, as well as doctors and other health professionals. Using a structural equation modeling, our results indicated that the predictors of patient-centered care for both administrators and those providing clinical care were participation in the accreditation process, leadership commitment to quality improvement, and measurement of quality improvement outcomes. In particular, perceiving the importance of the hospital’s engagement in the accreditation process was shown to be relevant to the administrators (gamma = 0.96), nurses (gamma = 0.80), as well as to doctors and other health professionals (gamma = 0.71). However, the administrator staff (gamma = 0.31) was less likely to perceive the influence of measuring the quality improvement outcomes on the delivery of patient-centered care than nurses (gamma = 0.59) as well as doctors and other health care providers (gamma = 0.55). From the nurses’ perspectives only, patient centeredness was found to be driven by building an institutional framework that supports quality assurance in hospital settings (gamma = 0.36). In conclusion, accreditation is a leading factor for delivering patient-centered care and should be on a hospital’s agenda as a strategy for continuous quality improvement.

## Introduction

During recent decades, issues regarding the quality of health care have increased considerably in many low- and middle-income countries, including Jordan. Health care providers and managers recognize the importance of successful and cost-effective patient outcomes. Evidence has shown that all processes and activities necessary for delivering health services need to be controlled using a total quality management (TQM) system.^1,2^ This system includes a series of interacting practices that aim to monitor, assess, and improve the quality of care, where the delivery of this care should be patient centered.^3^ Patient centeredness implies that all the parties involved in health care delivery need to consider the patient’s needs, preferences, and expectations, while ensuring that patient values guide all clinical decisions.^4-6^

In medical settings, adopting a person-centered approach in providing care is largely based on changing the way of thinking and performing activities, wherein the patients need to be treated not as a set of diagnoses or symptoms but as equal partners in planning, developing, and evaluating care.^2^ This requires the creation of a feeling of shared ownership for patients, emphasizing the importance of their participation in decision making and working alongside health care professionals to determine what matters to them and how to improve the quality of service delivery. Offering care in a more patient-centered way can ultimately contribute to satisfied and loyal patients, improved care delivery, and better clinical outcomes.^7,8^

In the literature, it has been widely acknowledged that accreditation is a key strategy to support best practices in assessing the quality of the provided care.^7,9,10^ Several studies have concluded that accreditation is a potentially effective tool for evaluating the compliance of health care organizations (HCOs) with pre-established standards,^11^ stimulate continuous quality improvement (CQI) strategies,^12,13^ and promote changes in the quality outcomes.^10,13-18^ Many authors have pointed out that the participation in the accreditation process can help hospitals create new leadership improvement initiatives^9,13^ and build robust systems for collecting and analyzing objective data on patient expectations, satisfaction, and/or complaints.^14,19,20^ While this may positively influence the quality of service delivery, the existing literature provides no clear evidence that accreditation can actually help in fulfilling the patients’ actual needs and expectations or in improving their level of satisfaction.^7,13,14^ Considering this, it is essential to examine the impact of applying different quality management (QM) practices on patient centeredness within the context of health care accreditation. The objectives of our study may therefore be stated as the development of a conceptual model of patient centeredness to identify the attributes that influence the delivery of patient-centered care (PCC), identification of the importance of these factors, and determination of whether there exist differences in the attributes identified by the administrative staff and health care providers. In particular, our study intended to answer the following research questions:

**Research Question 1:** What QM practices are associated with patient centeredness in hospital settings?
**Research Question 2:** What is the relative importance of the QM practices that influence patient centeredness in hospital settings?
**Research Question 3:** Are there any differences in the importance of QM practices that influence patient centeredness based on staff’s clinical and administrative functions in the hospital?

## Background

Similar to many countries in the Middle East (eg, Lebanon, United Arabs of Emirates, Saudi Arabia, etc.), Jordan is currently utilizing accreditation as a regulatory tool for ensuring the quality of health care and improving patient outcomes. In mid-2007, Jordan’s Ministry of Health (MoH), with the guidance of the US Agency for International Development and the Joint Commission International, launched the Jordan Health Accreditation Project (JHAP) as a national strategy for improving the quality of health care.^21^ JHAP’s vision was that “Jordan would have an agency in place that would continue to improve the quality and safety of health care services for all Jordanians after the project ended.”^21^ In keeping with this vision, JHAP has worked with the Health Care Accreditation Council (HCAC) to establish a fully functioning national accrediting agency. In March 2013, JHAP provided the HCAC with sufficient funds to operate its activities during the transition to an independent organization.

While the accreditation process is voluntary in Jordan,^22^ many HCOs consider it essential. Globally, larger organizations (eg, hospitals) are likely to value accreditation more than smaller facilities because the larger establishments are able to afford the potential costs associated with participation in such processes.^18,23,24^ In Jordan, there are 17 hospitals and specialist institutions currently accredited by the HCAC; only 5 of these hospitals belong to the MoH. Through the accreditation process, a team of authorized external peer reviewers conducts periodic on-site visits, generally every 2 years. During these visits, the survey team observes organizational processes; conducts interviews with managers, staff, and patients; and reviews the medical documentation pertaining to the adherence to a set of standards.

In the case of Jordan, there is still lack of compelling evidence for accreditation benefits and widespread critique that it is time-consuming and incurs additional costs. Findings from a recent research conducted in the country have indicated that Jordanian hospitals need to identify whether the participation in accreditation programs would affect the delivery of good quality patient-centered services.^25,26^

## Development of a Conceptual Model

This study presents a conceptual model that was developed based on the Baldridge quality recognition criteria as a CQI strategy. These criteria cover several aspects of QM, such as leadership and management, quality outcomes, management process of quality, as well as customer focus and satisfaction. Previous research has reported that the use of such criteria can enable a clearer picture of what a management paradigm should consider while assessing an organization’s progress toward meeting patients’ needs and expectations.^10^

**Figure 1.**
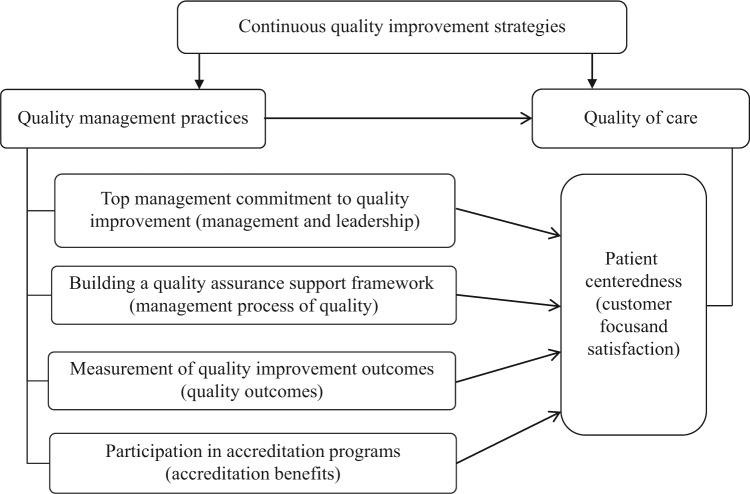
Conceptual model for the impact of quality management practices on patient centeredness.

Putting patients at the center of health care has also been recognized as a way to perform well on quality outcomes.^2^ Under this view, we argue that the implementation of the following 4 QM practices positively impacts patient centeredness: top management commitment to quality improvement (QI); building an institutional framework supporting quality assurance (QA); measuring and analyzing the outcomes of QI; and an organization’s participation in QA programs (eg, accreditation programs) (Figure 1). Based on the limited evidence, we also assumed that administrators hold a different view from clinicians and that nurses are more aware about the needs, circumstances, and preferences of the patients receiving care than other health care professionals. This assumption is supported by several studies,^24,27-31^ wherein the perceptions of nurses were targeted as an information source for determining the impact of QI and accreditation on the delivery of health care services. According to these studies, the role of the nursing staff is considered to contribute toward improvement in the quality of care and achievement of a better understanding of what is important to the patient. A large part of patient care is centered on the work of nurses who spend most of their work hours interacting with patients; therefore, logically, they are more likely than other health care providers to perceive the impact of the CQI strategies on the delivery of PCC.^29,32^

## Top Management Commitment to Quality Improvement

According to our model, health care leaders are in a prime position to integrate patient values and goals into all the aspects of management. Several studies have indicated that the leadership’s vision can play a primary role in supporting organizational commitment to improving the quality of patient experience and creating the right conditions and circumstances for PCC to flourish.^10,15,18,33-38^ This implies a style of leadership that truly focuses on encouraging and empowering the staff to change the services locally and work in a more patient-centered manner.^2^ To ensure the delivery of PCC, many experts, including Jack Silversin and Mary Kornack,^39^ have identified the importance of changing the culture within an organization from one focused on accommodating the physicians to that directed toward prioritizing the patient. While the role of leadership in driving changes and guiding all staff members is well documented in the literature,^20,32,40^ little is known about whether or how the executives’ engagement in QI activities can influence patient’s actual goals and expectations.

## Building a Quality Assurance Support Framework

In health care industry, the focus on patients’ actual needs is considered an important contributor to the overall success of TQM system.^41,42^ To achieve consistent, efficient, and sustainable improvement in the quality of care, it is necessary for HCOs to establish a QA infrastructure and develop policies that consider the patient’s values and expectations. It has been established by previous studies that incorporating QI activities in the daily operations of hospitals is a key factor for offering patient-centered services.^12,16,42^ In other words, if HCOs want to deliver PCC effectively, they must create and nurture an environment that embeds the QM principles into daily activities performed by all staff members. Calls for PCC have often emphasized the implementation of infrastructural changes regarding basic operational structures, conditions, policies, and relationships in health care. Practically, changes to promote PCC may involve a broad range of activities, including the exploration of patient’s values and preferences; involvement of patients and their families in care planning and decision making; facilitation of the access to appropriate care; engagement of patients in 2-way sharing of information; and obtainment of patient feedback on performance.^42^

## Measurement of Quality Improvement Outcomes

The extent to which patient expectations and satisfaction are met is a vital attribute that is considered while measuring the progress in quality outcomes.^4,19^ Therefore, before thinking about quality measurement, the priority should be to focus on the issues that patients value most rather than making assumptions regarding the things important to them.^2^ In health care QI, it is well-recognized that “you cannot manage what you cannot measure.”^42(p11)^ Thus, the presence of a robust system that enables an organization to monitor and measure the results of QI is essential to drive health system performance and maintain greater focus on customer needs. According to Shaller,^42^ the systematic measurement of QI and gathering of feedback have been identified as useful tools for assessing the effect of developing specific interventions and strategies on patient centeredness, including accreditation.

Other benefits that can result from routine measurement and reporting of quality data include comparing the best practices within an organization, identifying areas of improvement, and increasing the hospitals’ accountability toward different stakeholders (such as patients, families, government, or accreditation bodies).^32,41,42^ In this regard, it is noteworthy that accreditation is getting increasing attention as a possible approach to achieve better documentation of the quality processes, collect and analyze patients’ complaints/feedback data, report care outcomes, and regularly monitor an organization’s performance.^32,43-45^

## Participation in Quality Assurance Programs

While accreditation bodies are increasingly utilizing patient satisfaction surveys as a standard practice to assess the quality of care,^19,46^ the results of these surveys do not appear to have an influence on the accreditation decisions.^47,48^ In systematic reviews conducted in 2008 and 2014,^7,14^ researchers could not find any systematic or conclusive evidence to support the benefits of accreditation in enhancing patient satisfaction. According to a study by Sack et al,^49^ hospital accreditation represents a step toward QM; however, it is not a crucial factor contributing to patient satisfaction, as measured by their willingness to recommend the service to others. A Lebanese study conducted by Haj-Ali et al^16^ also reported a nonstatistically significant association between hospital accreditation classification and satisfaction as perceived by the patients. Their findings raise a concern about the importance of adopting an accreditation approach in hospitals to meet the patient’s expectations and goals and to achieve satisfaction. Considering that a majority of previous research has assessed the impact of the accreditation schemes on satisfaction from the patient’s perspective,^20,22,25^ considerable benefits can be obtained by tackling this issue from the viewpoint of the health care providers and administrators. Several studies have demonstrated that if accreditation strengthens the adherence to evidence-based standards and enhances commitment to best practice of QI, then it contributes to fulfilling patient’s desire for high-quality services.^32,50-54^

## Methods

### Study Design and Instrument

A multiple-case study design was used with a cross-sectional approach. According to Yin,^55,56^ evidence from multiple cases is often considered more compelling and allows the reinforcement of the data validity by providing more insights into the research. Within the literature on health care, very few instruments are available to examine the impact of applying the QM practices on patient centeredness, particularly within the context of accreditation. For our study, we used a structured pretested survey that was previously used in a Lebanese study conducted by El-Jardali et al^29^ to assess the perceived impact of accreditation on the quality of care among health care professionals. The questionnaire included the following 7 scales (composed of 54 items): management and leadership, strategic quality planning, human resource utilization, management process of quality, customer focus and satisfaction, quality outcomes, and benefits of hospital’s accreditation. With the exception of the last scale, all the scales were originally developed by Shortell et al^57^ based on the Baldridge quality recognition criteria. The scale on the benefits of hospital accreditation is adapted from a study conducted by Pomey et al^58^ to explore the dynamics of change that operated in hospital settings following participation in the accreditation process.

### Data Collection and Sampling Technique

The target population of this study was health care providers and administrators who worked at 4 accredited MoH hospitals. As mentioned earlier, the MoH has 5 accredited hospitals; however, one of these hospitals is small-sized (<100 beds). Employees who work at small hospitals may have a different scope of experience than those working in larger ones^29^; therefore, the small-sized hospital was excluded from the analysis. All the included hospitals had successfully passed 2 cycles of the national accreditation surveys (2011/2012-2013/2014).

A list of all the targeted staff members (eg, administrators, nurses, doctors, pharmacists, and allied health personnel) was obtained from the department of human resources at each of the participating hospitals. A staff member was eligible for study participation only if she/he had worked at the same hospital for the previous 3 years. This inclusion criterion was applied to ensure that the employees had participated at least in 1 of the 2 accreditation surveys and that he/she was familiar with and/or involved in the hospital’s accreditation process. After applying the previous inclusion criterion, our initial list was updated. Using a stratified sampling technique, we selected our sample by taking subgroups from each job category of interest. Within each stratum, simple random sampling was applied. By implementing such a technique, each staff member was chosen entirely by chance and had an equal chance of being included in the sample. Upon the participants’ approval, the survey forms were distributed to them at their departments. Respondents were assured that their participation was completely voluntary and that no one outside the study team would have access to the collected data. Participants were requested to complete a self-administrated questionnaire during their available time and return it in a sealed envelope within a week. The data collection period extended from September 2014 to December 2015. Of the total 919 staff members who were contacted, a sample of 829 clinician and nonclinician staff was successfully able to complete the survey, resulting in a response rate of 90%.

To explore the differences in the views of patient centeredness among the various health care workers, our data set of 829 respondents was divided into 3 groups to represent the study’s models. These groups consisted of 297 administrators (238 bureaucratic and 59 professionals with administrative responsibilities); 325 registered nurses; and 207 respondents, comprising 108 doctors and 99 other health care professionals, including pharmacists and allied health personnel, such as laboratory technicians and radiotherapists.

### Ethical Approval

Ethical approval to conduct this study was obtained from the Jordanian MoH Ethics Committee and the Institutional Review Board at the Jordan University of Science and Technology (192/2014).

### Data Analyses

An exploratory factor analysis with oblique promax rotation was conducted on the data set of 829 respondents to identify the distinct factors involved. The assumptions for such an analysis were met with Kaiser-Meyer-Oklin (KMO) test for sampling adequacy (KMO = 0.97 and *P* < .001). Using scree plot of explained variance by extracted factor, 5 factors covering 29 items were identified that cumulatively accounted for 60.5% of the variance. These factors were named as follows: patient centeredness (6 items), participation in accreditation programs (7 items), building a QA support framework (6 items), measurement of QI outcomes (5 items), and top management commitment to QI (5 items). Items were rated on a 5-point Likert scale ranging from 1 (strongly disagree) to 5 (strongly agree). The items loading on each factor are shown in Table 1. All included items had loadings of > 0.5 on their respective factors, reflecting good purification of constructs.^59^

**Table 1.** Rotated Factor Solution.

Variable number	Item description	Component				
		1	2	3	4	5
Patient centeredness						
1	The hospital assesses patients’ current needs and expectations	−0.056	−0.037	−0.043	**0.812**	0.178
2	The hospital assesses patients’ future needs and expectations	−0.119	−0.025	−0.017	**0.871**	0.150
3	The hospital resolves patient complaints	0.051	−0.092	0.025	**0.835**	−0.046
4	The hospital studies patient complaints to prevent the same problems from recurring	0.138	0.093	0.031	**0.712**	−0.149
5	The hospital communicates data on patient satisfaction to hospital staff	0.182	0.114	0.048	**0.582**	−0.102
6	The hospital uses data on patient expectations and/or satisfaction when designing new services	0.192	0.061	0.053	**0.586**	0.002
Top management commitment to quality improvement						
7	The top managers consistently participate in quality improvement activities	0.043	−0.008	**0.809**	0.056	−0.048
8	The top managers have a clear vision for improving the quality of care	−0.016	−0.055	**0.888**	0.072	−0.026
9	The senior executives have the ability to manage changes	0.054	0.002	**0.887**	−0.083	0.008
10	The senior executives have a thorough understanding of how to use accreditation results to improve quality	−0.044	0.043	**0.841**	−0.031	0.036
11	The senior executives generate confidence that efforts to improve quality will succeed	−0.035	0.049	**0.712**	0.023	0.065
Building a quality assurance support framework						
12	The hospital checks equipment and supplies for quality assurance purposes	0.016	**0.621**	0.045	0.116	−0.007
13	The hospital has effective policies to support quality improvement	−0.075	**0.811**	0.011	0.059	0.074
14	The hospital tries to introduce quality assurance into new services	0.085	**0.672**	0.029	0.048	0.059
15	The hospital tests services for quality assurance before they are implemented	0.031	**0.871**	−0.039	−0.022	0.017
16	The hospital views quality assurance as a continuing search for quality improvement	0.115	**0.832**	0.015	−0.076	−0.018
17	The hospital encourages staff to document quality problems	0.106	**0.669**	0.040	−0.040	0.054
Measurement of quality improvement outcomes						
18	Over the past 3 years, the hospital has shown steady, measurable improvements in customer satisfaction	0.084	0.065	0.005	0.056	**0.669**
19	Over the past 3 years, the hospital has shown steady, measurable improvements in the quality of services provided by the administration	0.076	0.077	0.001	0.009	**0.738**
20	Over the past 3 years, the hospital has shown steady, measurable improvements in the quality of clinical care provided to patients	0.038	−0.018	0.007	0.016	**0.875**
21	Over the past 3 years, the hospital has shown steady, measurable improvements in the quality of allied health services provided to patients	0.060	0.038	−0.006	0.011	**0.788**
22	Over the past 3 years, the hospital has maintained high-quality health services despite financial constraints	0.001	0.095	0.043	0.187	**0.580**
Participation in accreditation programs						
23	Accreditation enables the improvement of patient care	**0.677**	−0.036	0.027	0.030	0.193
24	Accreditation enables the development of values shared by all professionals	**0.768**	0.075	0.066	−0.071	0.051
25	Accreditation motivates staff and encourages teamwork and collaboration	**0.781**	0.038	0.035	−0.004	−0.016
26	Accreditation enables the hospital to better use its internal resources	**0.791**	0.114	−0.016	0.035	−0.029
27	Accreditation enables the hospital to better respond to patient needs	**0.766**	0.095	−0.068	0.112	−0.018
28	Accreditation contributes to collaboration with other partners	**0.776**	0.026	−0.015	0.061	−0.014
29	Accreditation enables hospitals to be more responsive when changes are to be implemented	**0.770**	−0.128	0.028	−0.018	0.148
Variance explained %		14.60	12.10	12.00	11.80	10.00
Cumulative percentage of variance (%)		14.60	26.70	38.70	50.50	60.50

In all the study’s models, the scale on patient centeredness constitutes the outcome variable, while the other factors represent the explanatory variables. This scale consists of several questions, including whether the hospital made sustained efforts to resolve the patients’ complaints and assess their current/future needs and expectations; if data on patient satisfaction are widely communicated to the staff and if the hospital uses such data when designing new services; and if the patients’ complaints are taken into consideration to identify patterns and prevent the same problems from recurring.

The conceptual model shown in Figure 1 was tested by structural equation modeling (SEM). This modeling technique is distinguished by its ability to make a clear distinction between a latent variable and its observable measures; to identify whether one path is more or less important than other paths in predicting the outcome measure; and to examine if two or more groups differ in their regression coefficients.^60^ The *lavaan* package of the R statistical computing software was used to fit all proposed models. All variables demonstrating a *P* value < .05 were considered statistically significant.

## Results

### Profiles of Respondents

Summary statistics for the sample population are presented in Table 2. Most participants were women (66.5%) and the age of 58.6% of the study population was between 30 and 45 years; 29.2% of them had been working at their respective hospital for 3 to 5 years. As shown in Table 2, majority of the respondents were nurses (39.2%), followed by administrators/bureaucratic (28.7%) and physicians (13.1%).

**Table 2.** Demographic Characteristics of the Study Participants by Occupational Category (n = 829).

Variable	Category			Total (N = 829)
	Administrators (n = 297)	Nurses(n = 325)	Doctors and other professionals (n = 207)	
	n (%)	n (%)	n (%)	n (%)
Gender				
Female	188 (63.30)	267 (82.15)	96 (46.38)	551 (66.5)
Male	109 (36.70)	58 (17.85)	111 (53.62)	278 (33.5)
Age				
<30	46 (15.49)	117 (36.00)	57 (27.54)	220 (26.6)
30-45	185 (62.29)	196 (60.31)	105 (50.72)	486 (58.6)
46-55	58 (19.53)	12 (3.69	32 (15.46)	102 (12.3)
>55	8 (2.69)	0 (0)	13 (6.28)	21 (2.5)
Years of experience in the hospital				
3-5	82 (27.61)	83 (25.54)	77 (37.20)	242 (29.2)
5.1-10	56 (18.86)	86 (26.46)	56 (27.05)	198 (23.9)
10.1-15	48 (16.16)	80 (24.62)	27 (13.04)	155 (18.7)
>15	111 (37.37)	76 (23.38)	47 (22.71)	234 (28.2)
Job				
Head of department/division	59 (19.9)	—	—	59 (7.1)
Administrator/bureaucratic	238 (80.1)	—	—	238 (28.7)
Registered nurse	—	325 (100)	—	325 (39.2)
Physician	—	—	108 (52.3)	108 (13.1)
Pharmacist	—	—	30 (14.4)	30 (3.6)
Allied health personnel(eg, laboratory technicians and therapists)	—	—	69 (33.3)	69 (8.3)

### Scale Construction: Unidimensionality

A confirmatory factor analysis was performed to confirm the unidimensionality of the measurement scales.^59^ The chi-square test was significant (*P* < .00) in all models; however, *P* value is sensitive to large samples; therefore, other fit indices were considered. As shown in Table 3, the model fit indices show that the values of the comparative fit index (CFI), normed fit index (NFI), and nonnormed fit index (NNFI) were > 0.95, indicating a very good model fit. Similarly, the values of the standardized root mean square residual (SRMR) and root mean square error of approximation (RMSEA) of ≤ 0.05 also show an acceptable model fit.^59,61^ Based on these threshold values, we concluded that the proposed factor structure for our models was valid.

**Table 3.** The Results of Confirmatory Factor Analysis.

Index	Category		
	Administrators (n = 297)	Nurses (n = 325)	Doctors and other health care professionals (n = 207)
Chi-squared	593.7	782.5	561.3
Degrees of freedom	367	367	367
Normed Chi-squared	1.618	2.132	1.529
*P* value	.000	.000	.000
CFI	0.9987	0.9977	0.9984
RMSEA	0.04568	0.0591	0.0507
NFI	0.9967	0.9958	0.9955
NNFI	0.9986	0.9975	0.9983
SRMR	0.0358	0.0393	0.0416

*Note.* CFI = comparative fit index; RMSEA = root mean square error of approximation; NFI = normed fit index; NNFI = nonnormed fit index; SRMR = standardized root mean square residual.

### Confirmation of Scale Reliability and Validity

To assess the internal consistency of each of the 5 study scales, composite reliability (CR) was measured using a value of 0.70 as a threshold.^59,61^ Convergent and discriminant validates were also measured to test the construct validity. Hair et al^59^ suggested that the average variances extracted (AVE) metrics for all path loadings should be ≥ 0.5 to confirm convergent validity. According to the previous threshold values, our proposed model is acceptable and shows excellent reliability and convergent validity (Table 4). To analyze the relationships between latent variables, the discriminant validity was assessed by comparing the AVE with the corresponding inter construct squared correlation estimates, as recommended by Hair et al.^59^ As illustrated in Table 4, the square root of the AVE values of all the constructs were greater than the inter construct correlations, indicating the discriminant validity of the constructs.

**Table 4.** Summary Statistics for Scale Items.

Factor	Item description	No. of items	CR	AVE				
				1	2	3	4	5
F1	Participation in accreditation programs	7	0.921	0.703				
F2	Building a quality assurance support framework	6	0.929	0.638	0.685			
F3	Top management commitment to quality improvement	5	0.924	0.462	0.511	0.710		
F4	Patient centeredness	6	0.921	0.640	0.635	0.491	0.660	
F5	Measurement of quality improvement outcomes	5	0.933	0.622	0.603	0.432	0.518	0.735

*Note.* CR = composite reliability; AVE = average variance extracted.

### Testing of Structural Models

Given that the unidimensionality, reliability, and validity were established for each of the measurement scales, it was appropriate to examine our proposed model.

### Administrative Staff (297 Cases)

**Figure 2.**
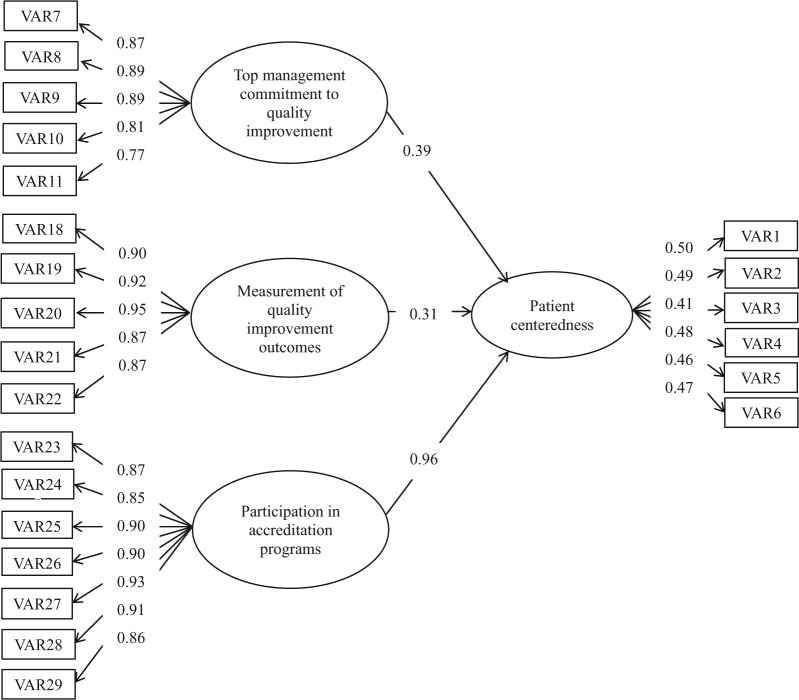
Path coefficients for administrators’ model.

According to the results of the SEM, the fit of the administrators’ model was acceptable. However, one path in the structural model was nonsignificant (path loading = 0.25, *P* value = .11). This one nonsignificant path, building a QA support framework, was therefore omitted. The reformulated measurement model was again tested using SEM, with 3 factors postulated to influence patient centeredness (Figure 2). These factors included hospital’s participation in the accreditation process (gamma = 0.96), top management’s commitment to QI (gamma = 0.39), and measurement of the QI outcomes (gamma = 0.31). The model fit was good and supported by a normed chi-square of 1.62, with the following fit indices: SRMR = 0.036; RMSEA = 0.046; NNFI = 0.99; CFI = 0.99 (Table 3).

### Nurses (325 Cases)

**Figure 3.**
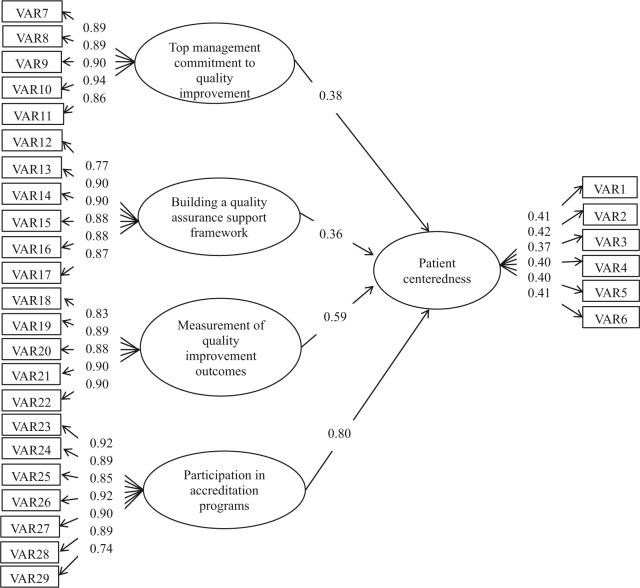
Path coefficients for the nurses’ model.

As shown in Table 3, the results of SEM demonstrated that the model fit was acceptable, and all paths in the model of nurses were significant (normed chi-square = 2.13; SRMR = 0.039; RMSEA = 0.059; CFI = 0.99). As per Figure 3, patient centeredness was influenced by the following factors: hospital’s engagement in the accreditation process (gamma = 0.80), measurement of the QI outcomes (gamma = 0.59), top management’s commitment to QI (gamma = 0.38), and building a QA support framework (gamma = 0.36).

### Doctors and Other Health Care Professionals (207 Cases)

**Figure 4.**
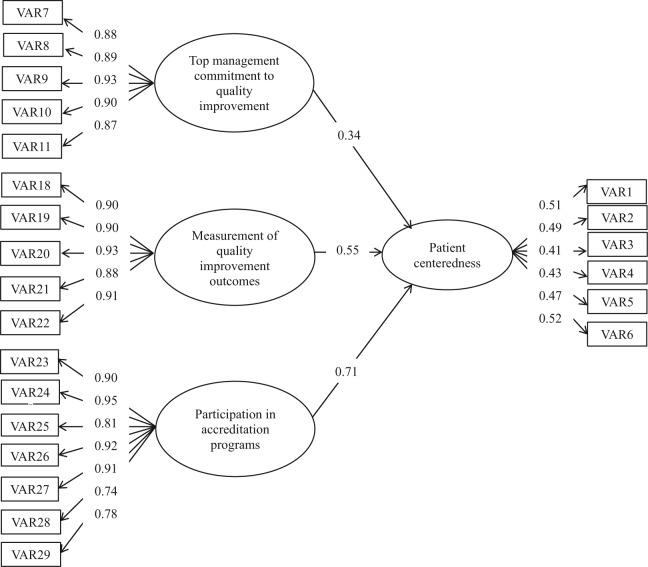
Path coefficients for the doctors and other health care professionals’ model.

While the fit of the doctors and other health care professional’s model was acceptable, one path in the structural model (building a QA support framework) was nonsignificant (path loading = 0.27, *P* value = .06). Consequently, this path was omitted, and the model was again tested using SEM. As Figure 4 indicates, patient centeredness is influenced by the following 3 factors: participation in the accreditation programs (gamma = 0.71), measurement of the QI outcomes (gamma = 0.55), and top management’s commitment to QI (gamma = 0.34). The model was found to fit the data well with normed chi-square = 1.53; SRMR = 0.042; RMSEA = 0.051; and CFI = 0.99 (Table 3).

The parameters’ estimates for the linkage between latent factors among the 3 models developed in this study are shown in Table 5. This table also shows 95% confidence intervals on the value of each parameter.

**Table 5.** The Parameters’ Estimates for the Linkage Between Latent Factors Among the Study’s Models.

Model	Factor	Est	SE	*z*	*P* value	95% CI
Administrators	Participation in accreditation programs	1.014	0.131	7.732	.000	(0.757-1.271)
	Measurement of quality improvement outcomes	0.305	0.105	2.905	.004	(0.099-0.511)
	Top management commitment to quality improvement	0.386	0.090	4.293	.000	(0.210-0.562)
Nurses	Participation in accreditation programs	0.799	0.137	5.833	.000	(0.530-1.067)
	Measurement of quality improvement outcomes	0.591	0.132	4.477	.000	(0.332-0.850)
	Building a quality assurance support framework	0.357	0.128	2.790	.005	(0.106-0.607)
	Top management commitment to quality improvement	0.376	0.094	4.016	.000	(0.192-0.559)
Doctors and other health care professionals	Participation in accreditation programs	0.709	0.116	6.118	.000	(0.482-0.936)
	Measurement of quality improvement outcomes	0.551	0.129	4.281	.000	(0.299-0.804)
	Top management commitment to quality improvement	0.344	0.105	3.266	.001	(0.138-0.550)

*Note.* CI = confidence interval.

## Discussion

The study used SEM to identify the impact of applying the following 4 QM practices on patient centeredness in hospital settings: top management’s commitment to QI; building an institutional framework that supports QA; measurement of the QI outcomes; and participation in the accreditation process. Moreover, our study aimed to determine whether there was a difference in the importance of previous attributes between the clinical and administrative staff in the hospital.

Overall, the following 3 areas were significant for both hospital administrators and those providing clinical care to enhance the delivery of PCC: leadership commitment to QI, participation in the accreditation process, as well as measurement and analyses of the QI outcomes. Regardless of the occupational category, our findings illustrated that the top management’s visibility, involvement, concerns for maintaining QI, and ability to drive cultural changes related to the quality of patient care were important in creating added value for the patients. This concurs with the available literature wherein several studies have reported that the substantial and strong vision of senior leadership can help place patients at the central focus of the organization and direct the staff members to carry this vision forward.^10,15,18,36,38,42^ Many authors have argued that it is the responsibility of the hospital directors to embrace the philosophy of PCC in health care mission, provide a strategic direction to achieve successful implementation of PCC, and work with other managers to create a quality infrastructure that aims at responding to the patient’s needs and concerns.^42,46,62^

In terms of the usefulness of hospital accreditation, our results demonstrated that accreditation was the most decisive factor in driving patient centeredness among all study groups. In descending order of importance, perceiving the benefits of the hospital’s participation in accreditation programs was relevant to administrators (gamma = 0.96), nurses (gamma = 0.80), and doctors and other health care professionals (gamma = 0.71). Relevant researches have shown that administrative staff and managers are most knowledgeable and heavily involved in hospital accreditation.^13,17^ While much accreditation paperwork is directly associated with clinical care, this managerial burden is usually borne by employees who hold administrative responsibilities. Technically, there is a high demand on the time of the administrative staff to implement activities related to obtain accreditation, including preparation of compliance reports and documents, working with surveyors, ensuring the accuracy of hospital records, and responding to data requests from accreditation bodies. Thus, we argue that the administrative/managerial staff is more aware about the importance of accreditation in increasing an organization’s focus on patient needs and expectations. This concurs with previous studies that viewed accreditation as a means to enhance the commitment to best practices of QI and increase the focus on quality patient care.^11,52,63^ However, other studies have reported several concerns of senior staff and managers about accreditation, including the bureaucratic and financial burden it imposed on health care facilities.^58,64-66^

In the health care industry, the clinicians’ perceptions toward accreditation are generally favorable.^12^ In multisite studies, nurses had supportive attitudes toward hospital accreditation in terms of its role in improving quality indicators and enhancing patient safety and satisfaction.^28,30,31^In a study by Aryankhesal,^67^ some head nurses in Iranian hospitals stated that increasing patient satisfaction was one of their incentives for obtaining a good accreditation grade.

With respect to the literature on physicians’ culture, mixed results have been obtained. According to an Australian study, a majority of physicians agreed that the accreditation process provided significant benefits to their organization.^68^ These benefits involved the promotion of commitment to best quality practices, improved structure for quality, and greater patient focus. Elsewhere, similar findings were also reported.^69^ In contrast, a number of studies have illustrated that physicians are skeptical about the importance of accreditation, raising concerns about its impact on the quality of health care services.^11,70^ In addition, Pomey et al^13^ and Touati and Pomey^71^ have noted a low tendency of the physicians to participate in QI programs (eg, accreditation). A qualitative study by Stoelwinder^70^ has also illustrated that the consistent, high workload in hospitals results in a situation where doctors feel more accountable within their professional framework, for example, to the patient and family, peers, and their profession, but not to the accreditation bodies. In other words, physicians may feel irritated if their role is shifted from that of giving priority to patients to one focusing on complying with the bureaucratic controls required by the accrediting organizations. Other health care professionals (eg, laboratory technicians) also viewed accreditation as a way to improve laboratory services by introducing more documentation and promoting better health and safety training procedures.^65^

Furthermore, measuring the QI outcomes was found to contribute to the enhancement of patient centeredness among all study groups. In particular, clinicians (eg, nurses) were more likely to perceive the influence of measuring the QI results on the delivery of PCC than the administrator staff. This is precisely what health care personnel with practical patient experience would logically expect. As is well known, nurses are responsible for a large part of patient care and are often responsible for a multitude of tasks that include direct contact with the patients. Considering this critical role, nurses have one of the greatest opportunities to assess the results of QI on attaining the patient’s goals and to monitor factors that might affect patient experience with care as an outcome of the service received. Our findings are consistent with those of other studies wherein several authors have argued that the measurement of quality outcome indicators strongly depends on the clinical frontline staff.^72-74^ Related studies have also indicated that the commitment and involvement of the hospital staff in measuring the quality indicators is associated with the success of the QI initiatives and better patients outcomes.^41,75^

The modeling of patient centeredness also presented an interesting finding. Strictly, administrator staff and health care professionals (eg, physicians, pharmacists, and therapists) do not perceive that developing a QA framework and supportive policies is important for patient centeredness. As per our study results, nurses reported that establishing a QA infrastructure that values patient goals and needs could be a method to provide care that is ultimately focused on the patient. As per the literature, the improvement of clinical care involves nurses as leaders and specialists in the formal quality activities rather than other professionals. It is obvious that several QA activities are assigned to the quality control entity, usually operated by the nursing staff; thereby, nurses have sound technical knowledge of the measures for improving the quality of patient experience.^13^ Considering this, moving toward a patient-centered approach requires the nurses to assume a leadership role in articulating the hospital’s commitment to meet the unique needs of the patients. To achieve this task, nursing leadership needs to identify the existing QM practice that can best serve in enhancing patient-centered infrastructure and find ways to meaningfully capture the relevant aspects of PCC across clinical contexts.

Alternatively, health care providers, such as physicians, do not receive enough training on QA methods and policies as part of their curriculum in medical schools and might be unaware of the essential approaches that can be used to support QI.^33,42^ This explains why Jordanian physicians did not perceive the impact of developing a QA framework and supportive policies on improving patient centeredness. Recent research have reported that the staff’s low engagement; tight schedule; disconnection with QI activities; and lack of awareness, familiarity, or knowledge concerning quality issues can lead to an underestimation of the importance of developing a QA framework that values patient’s goals.^18,41,76^ Another study supports this notion, showing that it is necessary for HCOs to identify an appropriate approach for raising the awareness of professionals to participate in adopting a patient-centered model in providing care.^13^

### Managerial Implications

Putting patients at the center of health care has profound implications for the way care is planned, provided, and evaluated. To enable effective PCC services, the hospital’s staffs need to re-frame their health care services, see things from the patients’ perspectives, and interact with patients to make changes in their experience of care.

According to JHAP,^21^ the health care delivery system throughout Jordan is still weakly regulated and has significant quality challenges. In this study, we developed a conceptual model that can be used by the directors of Jordanian hospitals to identify the QM practices influencing the delivery of PCC. Exploring the right conditions and circumstances for PCC to flourish would be a key step toward providing services capable of meeting the patient’s goals and expectations.

In the previous few decades, patients were expected to adjust with the routine practices that health care providers felt were most appropriate. This paternalistic approach where professionals “do things to” people may mismatch with the actual patient’s goals and may not be patient centered. However, for care to be enabling, the relationship between the patient and health care professionals needs to be based on the philosophy of “doing things with people,” rather than “to” them.^2^ Being inspired by this, there is an obvious need to enhance the awareness among health care providers, especially physicians, toward effectiveness and impact of building a culture of change to improve the patient’s experience with care.

As mentioned in the background section, hospital accreditation is still perceived by many Jordanian managers as a burdensome and costly process. Therefore, accreditation bodies (eg, HCAC) need to work closely with hospital directors to obtain their full support and involvement; help them permeate the relevant aspects of patient centeredness in all areas of care; focus on how to “do things differently” rather than requesting more things to do; and find ways to simplify the accreditation procedures and reduce the associated administrative burdens.

Traditionally, patients look for proof that the medical treatment they receive is of international standards. Hence, Jordanian MoH needs to launch social marketing campaigns aimed at targeting public awareness regarding the role of accreditation in reinforcing care outcomes. This approach would help regain the trust and confidence in public sector services. Furthermore, patients’ feedback surveys should be well planned and carefully address the domains that ensure responsiveness to patients’ preferences, needs, and values in addition to evaluating their “satisfaction.” The results of such surveys can be used as valuable sources for strategic planning in hospitals and to provide the necessary interventions.

A common theme resulting from the 3 models presented in this study was the positive impact of enhanced leadership commitment in improving service delivery. Leaders must demonstrate commitment by creating a seismic shift in the thinking with respect to empowering patients to take an active role in their care plan and responding to inputs from patients and families. Considering this, top managers of Jordanian hospitals are strongly encouraged to make patient centeredness a key aspect of their meetings and initiatives and actively engage staff members in open communication about things concerning the patients. By involving multidisciplinary groups of professionals, hospitals can derive a robust set of data from health care professionals who actually provide services to patients. Care providers often have their own disciplinary view of what the patient needs; thus, obtaining inputs from them can be meaningful and essential sources of data for addressing the needs and for developing an effective action plan for improving patient care.

Finally, the focus on establishing a QA infrastructure centered on patients and their values is a possible intervention that hospital managers and policy makers need to consider when addressing the different aspects of care. Researchers have concluded that the long-term success in QI requires changes in the attitudes of health care providers.^77^ In the case of Jordan, there is an obvious gap in the curriculum and the instructional mechanisms used in the schools of medicine in terms of inculcating the QA concepts into medical education. Thus, courses, teaching activities, and learning methods should be reviewed and revised on a continuous basis to ensure that sufficient information on QI is offered to students during their study.

### Limitations and Future Research

There are certain limitations to this study. Given that this study employed a cross-sectional design, causality cannot be ascertained. Longitudinal studies are necessary to compare the impact of applying QM practices on patient centeredness over time (eg, before and after accreditation). This could then be linked to a trend analysis aimed at assessing the differences in patient experiences after several years of accreditation. Another limitation is that the current study targeted only public hospitals affiliated to the Jordanian MoH. Thus, we cannot generalize these study findings to other hospitals in the country (eg, private sector hospitals). There is an obvious need for future larger scale studies. Social desirability bias can be a disadvantage of the self-reported questionnaires because participants often answer in a manner that portrays them in a good light. Future analyses would also be beneficial as they allow the exploration of how implementing QM practices may influence the delivery of PCC according to the patients’ perspectives. Additional studies that compare the various aspects of QM at accredited and nonaccredited hospitals are highly recommended.

## Conclusion

In keeping with the research objectives, our study identified several attributes that can help hospital managers choose appropriate practices for delivering patient-centered services. It was determined by clinical and nonclinical staffs that participation in the accreditation process is the most important factor that influences PCC in hospital settings. The administrative staff was less likely to perceive the influence of measuring the QI outcomes on the delivery of PCC than nurses, doctors, and other health care providers. From the nurses’ viewpoint, patient centeredness was driven by establishing a QA infrastructure and supportive policies that value patient’s goals.

Given that accreditation is a leading factor for delivering PCC, top managers and senior executives of Jordanian hospitals need to set accreditation on their agenda as a key strategy for influencing patient centeredness. Our findings offer opportunities for the Jordanian policy makers and hospital managers to learn practical strategies for implementing and sustaining PCC and to find areas that can be addressed to make an impact on care.

## Supplementary Material

Supplementary material
