# Supplementary material for: The Impact of Applying Quality Management Practices on Patient Centeredness in Jordanian Public Hospitals: Results of Predictive Modeling
Source: Inquiry. 2018 Feb 26;55:0046958018754739. doi: 10.1177/0046958018754739 (PMC5833210; doi:10.1177/0046958018754739)
Supplement: Supplementary material [file INQ_17_0031_R3_Supplementary_file.pdf]

Participants' responses for questionnaire items by occupational category

Variable #	Response	Occupational Category			p-value
		Administrators (n=297) %	Nurses (n=325) %	Doctors & other professionals (n=207) %	
V1: The hospital assesses patients' current needs and expectations	Strongly agree	11.45	9.85	6.76	0.005
	Agree	58.25	59.08	50.24	
	Neither disagree nor agree	11.78	13.54	24.15	
	Disagree	11.45	13.54	12.08	
	Strongly disagree	7.07	4.00	6.76	
V2: The hospital assesses patients' future needs and expectations	Strongly agree	5.39	4.62	4.35	0.002
	Agree	62.29	55.08	49.28	
	Neither disagree nor agree	11.78	20.31	28.99	
	Disagree	14.14	14.46	11.59	
	Strongly disagree	6.40	5.54	5.80	
V3: The hospital resolves patient complaints	Strongly agree	7.74	5.85	1.45	0.000
	Agree	53.20	49.23	39.13	
	Neither disagree nor agree	18.18	22.77	31.88	
	Disagree	12.46	17.23	17.87	
	Strongly disagree	8.42	4.92	9.66	
V4: The hospital studies patient complaints to prevent the same problems from recurring	Strongly agree	9.09	8.92	1.93	0.000
	Agree	54.55	49.54	41.55	
	Neither disagree nor agree	17.51	18.46	30.43	
	Disagree	11.45	16.92	15.46	
	Strongly disagree	7.41	6.15	10.63	
V5: The hospital communicates data on patient satisfaction to hospital staff	Strongly agree	7.41	8.31	2.42	0.000
	Agree	56.57	46.15	39.61	
	Neither disagree nor agree	17.17	21.23	24.15	
	Disagree	9.76	18.15	20.77	
	Strongly disagree	9.09	6.15	13.04	
V6: The hospital uses data on patient expectations and/or satisfaction when designing new services	Strongly agree	8.75	8.92	3.38	0.000
	Agree	57.24	50.15	42.51	
	Neither disagree nor agree	16.84	19.69	28.99	
	Disagree	10.44	17.85	16.43	
	Strongly disagree	6.73	3.38	8.70	

Participants' responses for questionnaire items by occupational category (Con't)

Variable #	Response	Occupational category			p-value
		Administrators (n=297) %	Nurses (n=325) %	Doctors & other professionals (n=207) %	
V7: The top managers consistently participate in quality improvement activities	Strongly agree	11.45	8.31	4.35	0.001
	Agree	54.88	55.08	46.38	
	Neither disagree nor agree	16.16	18.15	25.60	
	Disagree	10.44	15.38	16.43	
	Strongly disagree	7.07	3.08	7.25	
V8: The top managers have a clear vision for improving the quality of care	Strongly agree	10.44	6.46	2.90	0.000
	Agree	51.52	52.00	36.23	
	Neither disagree nor agree	16.50	16.62	33.82	
	Disagree	15.15	18.46	17.39	
	Strongly disagree	6.40	6.46	9.66	
V9: The senior executives have the ability to manage changes	Strongly agree	8.75	5.54	4.83	0.000
	Agree	48.15	51.69	35.75	
	Neither disagree nor agree	16.50	17.23	25.12	
	Disagree	16.50	20.92	23.19	
	Strongly disagree	10.10	4.62	11.11	
V10: The senior executives have a thorough understanding of how to use accreditation results to improve the quality	Strongly agree	10.44	5.85	3.38	0.002
	Agree	56.90	52.00	45.41	
	Neither disagree nor agree	15.49	19.38	24.64	
	Disagree	10.10	15.69	17.87	
	Strongly disagree	7.07	7.08	8.70	
V11: The senior executives generate confidence that efforts to improve quality will succeed	Strongly agree	11.45	7.38	6.28	0.031
	Agree	57.91	55.38	48.79	
	Neither disagree nor agree	15.15	16.00	21.26	
	Disagree	9.76	16.31	15.94	
	Strongly disagree	5.72	4.92	7.73	
V12: The hospital checks equipment and supplies for quality assurance purposes	Strongly agree	8.08	3.69	3.38	0.000
	Agree	52.86	56.00	43.96	
	Neither disagree nor agree	19.19	16.62	29.95	
	Disagree	13.47	20.00	14.98	
	Strongly disagree	6.40	3.69	7.73	

Participants' responses for questionnaire items by occupational category (Con't)

Variable #	Response	Occupational Category			p-value
		Administrators (n=297) %	Nurses (n=325) %	Doctors & other professionals (n=207) %	
V13: The hospital has effective policies to support quality improvement	Strongly agree	4.71	2.77	2.42	0.000
	Agree	59.26	58.15	46.86	
	Neither disagree nor agree	16.50	14.46	29.47	
	Disagree	13.47	19.69	13.53	
	Strongly disagree	6.06	4.92	7.73	
V14: The hospital tries to introduce quality assurance into new services	Strongly agree	12.12	7.69	3.38	0.000
	Agree	52.53	48.92	43.96	
	Neither disagree nor agree	19.87	21.23	28.99	
	Disagree	9.09	17.85	14.49	
	Strongly disagree	6.40	4.31	9.18	
V15: The hospital tests services for quality assurance before they are implemented	Strongly agree	10.44	6.46	3.38	0.000
	Agree	55.89	56.92	47.34	
	Neither disagree nor agree	18.86	18.77	26.09	
	Disagree	7.74	14.77	13.53	
	Strongly disagree	7.07	3.08	9.66	
V16: The hospital views quality assurance as a continuing search for quality improvement	Strongly agree	7.74	5.54	2.42	0.000
	Agree	60.94	55.38	51.69	
	Neither disagree nor agree	17.51	19.38	23.19	
	Disagree	8.08	16.31	12.56	
	Strongly disagree	5.72	3.38	10.14	
V17: The hospital encourages staff to document quality problems	Strongly agree	11.11	5.23	1.45	0.000
	Agree	52.19	55.08	44.93	
	Neither disagree nor agree	19.19	20.92	28.99	
	Disagree	10.44	14.46	15.46	
	Strongly disagree	7.07	4.31	9.18	
V18: The hospital has shown measurable improvements in customer satisfaction	Strongly agree	11.78	5.23	5.31	0.000
	Agree	58.25	58.77	43.96	
	Neither disagree nor agree	13.47	16.62	24.15	
	Disagree	9.09	13.54	14.01	
	Strongly disagree	7.41	5.85	12.56	

Participants' responses for questionnaire items by occupational category (Con't)

Variable #	response	Occupational category			p-value
		Administrators (n=297) %	Nurses (n=325) %	Doctors & other professionals (n=207) %	
V19: The hospital has shown measurable improvements in the quality of services provided by the administration	Strongly agree	7.07	4.92	1.93	0.003
	Agree	51.18	52.31	39.61	
	Neither disagree nor agree	14.81	14.46	24.15	
	Disagree	15.15	17.54	18.84	
	Strongly disagree	11.78	10.77	15.46	
V20: The hospital has shown measurable improvements in the quality of clinical care provided to patients	Strongly agree	7.07	3.38	2.42	0.004
	Agree	55.22	52.31	42.51	
	Neither disagree nor agree	16.50	19.69	28.99	
	Disagree	14.48	17.23	18.84	
	Strongly disagree	6.73	7.38	7.25	
V21: The hospital has shown measurable improvements in the quality of allied health services provided to patients	Strongly agree	7.74	4.31	3.86	0.001
	Agree	50.17	48.92	34.78	
	Neither disagree nor agree	17.85	17.85	28.50	
	Disagree	15.49	21.54	22.71	
	Strongly disagree	8.75	7.38	10.14	
V22: The hospital has maintained high-quality health services despite financial constraints	Strongly agree	7.07	4.31	1.93	0.001
	Agree	58.59	58.15	45.41	
	Neither disagree nor agree	16.16	19.08	26.57	
	Disagree	12.12	14.46	17.87	
	Strongly disagree	6.06	4.00	8.21	
V23: Accreditation enables the improvement of patient care	Strongly agree	6.06	4.00	1.93	0.001
	Agree	53.20	52.00	40.58	
	Neither disagree nor agree	18.52	20.92	27.54	
	Disagree	13.47	19.08	19.32	
	Strongly disagree	8.75	4.00	10.63	
V24: Accreditation enables the development of values shared by all professionals	Strongly agree	5.72	1.54	2.90	0.000
	Agree	52.86	54.77	42.51	
	Neither disagree nor agree	15.15	16.62	22.71	
	Disagree	16.50	21.54	17.39	
	Strongly disagree	9.76	5.54	14.49	

Participants' responses for questionnaire items by occupational category (Con't)

Variable #	Response	Occupational Category			p-value
		Administrators (n=297) %	Nurses (n=325) %	Doctors and other professionals (n=207) %	
V25: Accreditation motivates staff and encourages teamwork and collaboration	Strongly agree	10.10	8.62	2.90	0.001
	Agree	53.87	50.77	45.89	
	Neither disagree nor agree	19.19	17.85	29.95	
	Disagree	11.45	17.54	13.04	
	Strongly disagree	5.39	5.23	8.21	
V26: Accreditation enables the hospital to better use its internal resources	Strongly agree	6.73	6.77	3.86	0.001
	Agree	53.20	43.38	38.16	
	Neither disagree nor agree	17.17	21.85	30.43	
	Disagree	17.51	23.08	18.36	
	Strongly disagree	5.39	4.92	9.18	
V27: Accreditation enables the hospital to better respond to patient needs	Strongly agree	8.42	5.23	1.93	0.000
	Agree	49.49	48.00	36.23	
	Neither disagree nor agree	18.52	19.69	28.99	
	Disagree	17.85	22.15	21.26	
	Strongly disagree	5.72	4.92	11.59	
V28: Accreditation contributes to collaboration with other partners	Strongly agree	5.72	7.08	2.90	0.002
	Agree	56.57	45.23	43.00	
	Neither disagree nor agree	14.48	21.54	26.09	
	Disagree	17.51	20.92	18.36	
	Strongly disagree	5.72	5.23	9.66	
V29: Accreditation enables hospitals to be more responsive when changes are to be implemented	Strongly agree	4.04	6.46	4.83	0.005
	Agree	57.58	46.15	44.44	
	Neither disagree nor agree	13.47	19.69	23.67	
	Disagree	17.17	20.92	14.98	
	Strongly disagree	7.74	6.77	12.08	
